# Quality of perinatal care services from the user’s perspective: a Dutch study applies the World Health Organization’s responsiveness concept

**DOI:** 10.1186/s12884-017-1464-8

**Published:** 2017-09-29

**Authors:** Jacoba van der Kooy, Erwin Birnie, Nicole B. Valentine, Johanna P. de Graaf, Semiha Denktas, Eric A. P. Steegers, Gouke J. Bonsel

**Affiliations:** 1 Division of Obstetrics & Prenatal Medicine, Department of Obstetrics and Gynaecology, Erasmus MC, PO Box 2040, 3000 CA Rotterdam, The Netherlands; 2 World Health Organization, Avenue Appia 20, 1211 Geneva, Switzerland; 3 Department Obstetrics & Gynaecology, Academic Collaboration Maternity Care Services, University Medical Center Utrecht, PO Box 85500, 3508 GA Utrecht, the Netherlands; 4 Rotterdam Midwifery Academic (Verloskunde Academie Rotterdam), Dr. Molewaterplein 40, 3015 GD Rotterdam, The Netherlands; 5 Department of Public Health, Erasmus MC, PO Box 2040, 3000 CA Rotterdam, The Netherlands

**Keywords:** Quality of care, Responsiveness, Perinatal care, Birth care

## Abstract

**Background:**

The concept of responsiveness was introduced by the World Health Organization (WHO) to address non-clinical aspects of service quality in an internationally comparable way. Responsiveness is defined as aspects of the way individuals are treated and the environment in which they are treated during health system interactions.

The aim of this study is to assess responsiveness outcomes, their importance and factors influencing responsiveness outcomes during the antenatal and delivery phases of perinatal care.

**Method:**

The Responsiveness in Perinatal and Obstetric Health Care Questionnaire was developed in 2009/10 based on the eight-domain WHO concept and the World Health Survey questionnaire. After ethical approval, a total of 171 women, who were 2 weeks postpartum, were recruited from three primary care midwifery practices in Rotterdam, the Netherlands, using face-to-face interviews. We dichotomized the original five ordinal response categories for responsiveness attainment as ‘poor’ and good responsiveness and analyzed the ranking of the domain performance and importance according to frequency scores. We used a series of independent variables related to health services and users’ personal background characteristics in multiple logistic regression analyses to explain responsiveness.

**Results:**

Poor responsiveness outcomes ranged from 5.9% to 31.7% for the antenatal phase and from 9.7% to 27.1% for the delivery phase. Overall for both phases, ‘respect for persons’ (Autonomy, Dignity, Communication and Confidentiality) domains performed better and were judged to be more important than ‘client orientation’ domains (Choice and Continuity, Prompt Attention, Quality of Basic Amenities, Social Consideration). On the whole, responsiveness was explained more by health-care and health related issues than personal characteristics.

**Conclusion:**

To improve responsiveness outcomes caregivers should focus on domains in the category ‘client orientation’.

**Electronic supplementary material:**

The online version of this article (10.1186/s12884-017-1464-8) contains supplementary material, which is available to authorized users.

## Background

The performance of perinatal care is often judged by endpoints such as perinatal morbidity, mortality and costs. However, quality of care literature supports the view that non-clinical aspects of health care, such as service quality, are important aspects of the system’s performance too and, moreover, may affect clinical outcomes [1–3]. Better service quality is thought to increase compliance with medical treatment, and to improve information transfer and utilization of health services [4–7]. Governments of Western countries increasingly acknowledge the importance of incorporating non-clinical service quality when the performance of the system is monitored [8, 9].

An important approach to measuring service quality is the concept of ‘responsiveness’, which was introduced by the World Health Organization in the World Health Report 2000 to compare service quality in an internationally comparable way. Responsiveness is defined as aspects of the way individuals are treated and the environment in which they are treated during health system interactions [10]. Aspects refer to non-financial, non-clinical qualities of care that reflect respect for human dignity and interpersonal aspects of the care process. Being based on utility theory, the concept separates the utility individuals derive from clinical and non-clinical aspects, and from a policy perspective can be used to make trade-offs between non-clinical quality and clinical quality. Utility theory refers to the measurement of preferences over some set of goods and services [10]. Human rights law argues that the responsiveness features of a health system are important in their own right [10–12].

Perinatal care in the Netherlands is organized as a system of inter-related services, that include referral practices, covering the different phases of the perinatal experience: antenatal care, delivery and postpartum care. Perinatal care is provided by independently operating community midwives providing care for low-risk pregnant women (primary healthcare) and obstetricians and gynecologists providing in-hospital care for high-risk women (secondary and tertiary care). Most women receive postpartum care by a community midwife. Most perinatal deaths occur during the antenatal and delivery phases [13]. International studies and the National Report of the Netherlands reported that the perinatal mortality rate in 2004 for the Netherlands was the highest in Europe (10.5/1000 live births). In 2010 the perinatal mortality rate declined (9.0/1000 live births) [14–17]. As a result, the evaluation of the different aspects of perinatal care, in particular the antenatal and delivery phases, is crucial [18]. Evaluation of non-clinical aspects of the quality of care may be even more important, since the majority of women are not ill. This may increase the importance of non-clinical aspects. Thus far, few attempts have been made to evaluate non-clinical quality of the perinatal health care system across the different phases. The few studies available observe that aspects of health care services influence patient satisfaction [19–21]. They did not investigate the different phases of the perinatal system nor use an internationally comparable questionnaire. To our knowledge, ours is the first study to present this information for perinatal care in the Netherlands. The aim of our study was to assess the responsiveness outcomes and factors influencing responsiveness outcomes of perinatal health care in urban settings in the Netherlands using the Responsiveness in Perinatal and Obstetric Health Care Questionnaire, the ReproQ questionnaire, which was based on the WHO concept of health system responsiveness and modified from existing WHO questionnaires.

## Methods

### Questionnaire

The Responsiveness in Perinatal and Obstetric Health Care Questionnaire (ReproQ) was developed between October 2009 and February 2010. The ReproQ is based on the same eight domains identified for measuring responsiveness in WHO’s review of the patient satisfaction and quality of care literature. The eight domains were Dignity, Autonomy, Confidentiality, Communication (collectively categorized as the ‘respect for persons’ domains), Choice and Continuity, Prompt Attention, Quality of Basic Amenities, and Social Consideration (collectively categorized as ‘client orientation’ domains). To build the ReproQ, slight adaptations were made to the set of responsiveness questions translated from the WHO questionnaires [10, 22].

The ReproQ was designed, as with most of the WHO questionnaires, to be administered in a face-to-face interview setting. The ReproQ asks essentially the same set of questions for the three different phases of perinatal care but, for purposes of this paper, we focus on two phases - the antenatal and delivery phases – the most important for the infant mortality challenge mentioned earlier. More importantly, postpartum care is different in its characteristics and delivery site since it is delivered only at home and includes only home nurses and midwives limiting discussion of referral practices. In addition it includes evaluation of pediatric care. Data on the postnatal care will therefore be studied separately. The antenatal phase was defined as the period from the onset of pregnancy until the onset of delivery. Respondents were asked to provide an overall evaluation of their experiences that took place during the antenatal care period rather than just a single visit that may be biased in either way by a particular incident. The delivery phase referred to the period of birth.

Each phase was covered by the above mentioned eight domains, with 2–7 question items per domain. The standardized response options consisted of five verbal response categories: ‘very good’ , ‘good’ , ‘moderate’ , ‘bad’ , and ‘very bad’. In total, 65 responsiveness question items were distributed over two phases (25 antenatal, 40 delivery). In addition, 29 question items on personal and healthcare-related characteristics associated with the experience were also included.

Table 1 shows the eight domains and question items for the antenatal phase. The domains and items for the delivery phase were roughly similar. The following psychometric properties of the ReproQ were evaluated: feasibility, reliability and validity. Feasibility: the interviews lasted between 20 and 40 min and the overall missing rate was 8%. Construct validity: mean Cronbach’s alphas for the antenatal, birth and postpartum phase were: 0.73 (range 0.57–0.82), 0.84 (range 0.66–0.92), and 0.87 (range 0.62–0.95) respectively. The item-own scale correlations within all phases were considerably higher than most of the item-other scale correlations. Within the antenatal, birth and postpartum phase, the eight factors explained 69%, 69%, and 76% of variance respectively. Discriminative validity: overall responsiveness mean sum scores were higher for women whose children were not admitted, as expected from literature. The ReproQ interview-based questionnaire demonstrated satisfactory psychometric properties, with the potential to discriminate between quality of care levels. Detailed description on the reported psychometric properties are reported elsewhere [23].

**Table 1:** The eight domains with the question items formulated for the antenatal phase

Respect for persons	
Autonomy	How well were you involved in making decisions regarding your examinations or treatments?
	Were you able to refuse examinations or treatments?
	Were you asked permission before testing or starting treatment?
Dignity	Were physical examinations and treatments done in a way that respected your privacy?
	Did the examination rooms ensure your privacy?
	Were you treated with respect by your health care provider?
Communication	How well were things explained by your health care provider in a way you could understand?
	Was written information provided in such a way you could understand?
	Were you encouraged to ask questions about your health problems, treatment and care?
	Were you given time to ask questions about your health problem or treatment?
	Was information on the health service’s contact, location and parking information clear to you?
Confidentiality of Information	Were consultations carried out in a manner that protected your confidentiality?
	Was confidentiality kept on the information provided by you?
	Was your medical record kept confidential?
Client orientation	
Choice and Continuity of Health Care Provider	Were you able to choose your own health care provider?
	Were you able to use other health care services other than the one you usually went to?
	How well was the continuity of care by one health care provider?
	Were you able to choose your own place of delivery?
Prompt Attention	How well did you receive prompt attention at your health service?
	How did you experience the waiting time after you asked for help?
	How well was the accessibility by phone?
	How do you rate the travel time to your health service?
Quality of basic amenities	How do you rate the quality of the hygiene of the toilets?
	How do you rate the overall quality of the surroundings, for example, space, seating, fresh air and cleanness?
	How do you rate the quality of the food?
Social Consideration	Did the health care provider facilitate the support of your relatives and friends?
	Was the home situation taken into consideration when planning an appointment?

### Study population; data collection

The study was a cross-sectional, interview-based survey of women having had a delivery in the previous 2 weeks. Study approval was granted by the Medical Ethical Committee, Erasmus Medical Centre, Rotterdam, the Netherlands, no MEC2012207. Study respondents were recruited from three primary care midwifery practices in the urban area of Rotterdam, the Netherlands, between February 2010 and March 2011. These three practices were geographically chosen since they provide care for almost all women living at the north side of Rotterdam. Within these three midwife practices 25 different community midwives provide care. Women or their partners or family were required to speak and understand Dutch sufficiently, the latter serving as translators rather than proxy respondents. Written informed consent was obtained prior to the interview. Study interviews were carried out by 10 trained and independent interviewers, but first invitations to participate in the study were made by the respondents’ own midwife at the postpartum visit 2 weeks after delivery. Respondents were invited in a consecutive order, using the day of delivery. The interview was usually held at the home of the respondent. Interviewees were invited to respond to all questions, yet never forced to. The average interview was 30 min long.

### Responsiveness measures and background characteristics

Two responsiveness outcome measures were estimated to describe performance: question and domain measures. For question measures, the five options answers were grouped into binary categories; ‘good’ and ‘poor’. The ‘poor’ rating was used when a respondent reported the item as either ‘very bad’, ‘bad’ or ‘moderate’. For domain measures, if over 33% of the items were rated poor within a domain, the rating of ‘poor’ was used for the whole domain. The percentage approach was used to score domains as the number of question items per domain differed across domains. Dichotomization was chosen as it has been shown to reduce bias caused by reporting scale contraction for disadvantaged groups. Relevant differences in non-optimal outcomes can therefore be missed. For similar reasons and for reasons of the right-skewedness of the data, we chose to judge a domain as poor when at least 33% of the items were judged as poor [24, 25]. Dichotomization avoids problems associated with violating regression assumptions when testing which personal or health service factors are associated with responsiveness. Another commonly used metrics, sum scores, were positively skewed but we chose not to use this metric due to it being less useful for addressing reporting behaviour. Thresholds were selected ex ante and results were presented in the same way the WHO Responsiveness reports were presented in the past (http://www.who.int/responsiveness/papers/MCSS_Analytical_Guidelines.pdf).

Responsiveness domain importance measures were calculated based on individual rankings of the set of domains.

Background characteristics with a feasible influence over the responsiveness performance rating were chosen. These were: parity (nulliparous/multiparous), age (≤30/>30 years), ethnicity (Dutch/non-Dutch), education level (low or middle/high), marital status (single/relationship or married), living in a deprived neighborhood (yes/no, based on 4-digit zip-codes and a public list of deprived zip-code based neighbourhoods issued by the Dutch government) [26], Dutch language proficiency (good/weak or poor), obstetric history (yes/no, based on self-report of mother or child outcomes which required a medical intervention by a gynaecologist), adverse child outcome (yes/no, based on self-reported asphyxia, (possible) congenital anomaly, infection, small for gestational age (child too small), and/or premature birth), paediatric hospital admission within 1 month (yes/no), receiving pain medication when requested (yes/no), receiving an intervention (yes/no, instrumental delivery or a caesarean section), maternal hospital admission during the antenatal period or within 1 month after birth (yes/no), day of delivery (weekend/weekday), time of delivery (8-18 h/18-8 h), healthcare pathway during pregnancy (referral to secondary care during antenatal or birth care, yes/no), perinatal healthcare pathway (Start antenatal care with midwife, not referred; Start antenatal care with midwife, referred during antenatal care to gynaecologist; Start antenatal care with midwife, referred during birth care to gynaecologist; Antenatal and birth care with gynaecologist).

### Data handling

Records of a respondent were regarded missing if all scores on all phases were missing (antenatal, delivery and postpartum phase). If response was partial, the response was evaluated per phase. Respondents were excluded for one phase if all items were missing for that phase. Values for missing question items were imputed with the mean when only up to 3 items were missing in a particular phase. We imputed these values to increase precision and power. We imputed with the mean as a conservative approach. Bias is toward non-significance, hereby not overestimating assocations in our dataresults [27, 28]. Variables with over 30% missing values were not imputed and excluded from analysis.We assumed a baseline proportion of poor performance per domain of 10% (i.e. 90% of respondents has sufficient score); we further assumed that the difference between non-referred patients during delivery, and referred patients was substantial and relevant, expecting from several sources doubling i.e. 20% poor performers (see e.g. Rijnders 2008 [29]. Under these assumptions *n* = 196 is sufficient to discriminate between these known groups. Higher rates in general (as we actually observed in half of the domains) lower the sample size, smaller difference increase the size. Our final sample size was 171 respondents, implying that our analysis was slightly underpowered for the assumptions.

### Analysis

#### Descriptive analyses

Data were analysed using SPSS software version 17.0. Responsiveness performance and importance outcomes were described in frequency tables and by spider diagrams by phase. The assigned importance of each domain was plotted against domain scores (% good responsiveness) and visually inspected for any observed relationship.

#### Bivariate analyses

Spider diagrams were also used to show patterns in responsiveness outcomes between advantaged and disadvantaged subpopulations as was done in the WHO [30]. For these comparisons we grouped a set of disadvantaged subpopulations according to the following background characteristics: for ‘respect for persons’ than for ‘client orientation’ domains. In the antenatal phase, (1) multiparous, (2) Dutch-origin, (3) having started with a midwife, and not being referred, and (4) having no child hospitalization. The unpaired Student’s t-test or the Chi square test were used to compare groups on these characteristics.

#### Regression analyses

Multiple regression was used to explain responsiveness for each domain by the background characteristics (personal and healthcare-related).

Forward stepwise analysis was used (inclusion *p* < 0.05; exclusion *p* > 0.05) to explain domain outcomes.

## Results

A total of consecutive 274 respondents were invited for participation, 180 respondents (66%) agreed to be interviewed. Reasons for non-participation included the anticipated time burden, feeling at unease having a stranger visit their home, and logistic reasons such as incorrect phone number, or incorrect address. Of the 180 interviews planned, seven interviews (4%) were cancelled by the women and two interviews (1%) were cut short because the respondent’s language proficiency was inadequate and no translator was present. The remaining 171 interviews (95%) were analysed. 18(11%) of these interviews were conducted with either translation or in English.

Table 2 describes the respondent’s background characteristics. About 70% of women were between the ages of 25 and 34, only 4% had no or low education, half were of Dutch origin and about half came from underprivileged neighbourhoods, and most had a high proficiency of spoken Dutch (89%). Related to health-care characteristics, 60% were primiparous. On the perinatal health care pathway, about a third started antenatal care with a midwife and were not referred, and another third started antenatal care with midwife, and were referred during birth care to a gynaecologist.

**Table 2:** Respondent’s characteristics, obstetric outcomes and health care characteristics (*n* = 171)

	Number	Percent
Personal characteristics		
Maternal Age^a^		
< 19 years	3	2%
20 – <25 years	15	9%
≥ 25 – ≤ 34 years (REF)	119	70%
≥ 35 years	33	19%
Missing	1	1%
Parity		
Primiparous	97	57%
Multiparous (REF)	74	43%
Education		
Low	6	4%
Middle	75	44%
High (REF)	90	53%
Marital status		
Single	30	18%
Relationship/married (REF)	141	82%
Ethnic background		
Dutch (REF)	94	55%
Non Dutch	77	45%
Neighbourhood		
Privileged neighbourhood (REF)	84	49%
Underprivileged neighbourhood	87	51%
Proficiency (speaking) Dutch		
Good/excellent (REF)	153	89%
Weak/poor	18	11%
Health care-related characteristics		
Obstetric history^b^		
Primiparous	97	57%
Multiparous, no medical history (REF)	24	14%
Multiparous, medical history	50	29%
Perinatal health care pathway		
(1) Start antenatal care with midwife, not referred (REF)	61	36%
(2) Start antenatal care with midwife, referred during antenatal care to gynaecologist	37	22%
(3) Start antenatal care with midwife, referred during birth care to gynaecologist	57	33%
(4) Antenatal and birth care with gynaecologist	16	9%
Pain medication during labour		
No request (REF)	79	46%
No pain medication received after requesting	32	19%
Pain medication received after requesting	58	34%
Intervention during labour^c^		
No (REF)	97	57%
Yes, no emergency intervention	51	30%
Yes, emergency intervention	21	12%
Day of delivery		
Weekend	37	22%
Weekday (REF)	134	78%
Time of delivery		
0-8 h	45	26%
8-18 h (REF)	82	48%
18-24 h	43	25%
Missing	1	1%
Adverse outcome of child^d^		
No adverse outcome (REF)	128	75%
Adverse outcome	43	25%
Hospital admission of child		
No admission (REF)	145	85%
Admission	26	15%
Hospital admission of the mother		
No admission (REF)	154	90%
Admission	17	10%

*REF* reference in logistic regression

^a^Mean age 30 (range 18–42)

^b^Obstetric history based on self reported mother or child outcomes which required intervention of a gynaecologist

^c^Ceasarean section or instrumental delivery

^d^Adverse outcome based on self reported asphyxia (shortage of oxygen), (possible) congenital anomaly, infection, small for gestational age (child too small), premature birth

Table 3 shows the responsiveness performance outcome measures by domain for the antenatal and the delivery phases. The proportion of poor responsiveness outcomes ranged from 5.9% (Dignity) to 31.7% (Social Consideration) in the antenatal phase and from 9.7% (Dignity) to 27.1% (Choice and Continuity) in the delivery phase. For both phases, ‘respect for persons’ (Autonomy, Communication, Confidentiality, Dignity) domains were judged better than the ‘client orientation’ domains.

**Table 3:** Client reported poor responsiveness for each domain, for the antenatal and delivery phase separately

Domain	Antenatal phase		Delivery phase	
	Number of participants	Percentage reporting poor responsiveness	Number of participants	Percentage reporting poor responsiveness
Respect for persons				
Autonomy (AU)	161	18.0%	155	15.7%
Dignity (DI)	169	5.9%	165	9.7%
Communication (CM)	168	20.0%	166	14.2%
Confidentiality (CF)	159	7.8%	153	11.6%
Client orientation				
Choice and Continuity (CC)	167	28.1%	162	27.1%
Prompt Attention (PA)	169	30.0%	144	20.6%
Quality of Basic Amenities (QA)	168	22.9%	156	23.4%
Social Consideration (SC)	164	31.7%	158	22.1%

Domain importance measures were higher (higher frequency), for the ‘respect for persons’ domains than the ‘client orientation’ domains (average 69%; 95% CI 60%–76% versus 31%; 95%CI 24%–40%). The highest importance was assigned to the domains of Communication (26%) and Dignity (22%) and the lowest was assigned to Choice and Continuity (6%) and Social Consideration (4%). Of similar importance were Autonomy, Confidentiality, Prompt Attention, and Quality of Basic Amenities (range: 10%–11%).

**Fig. 1:**
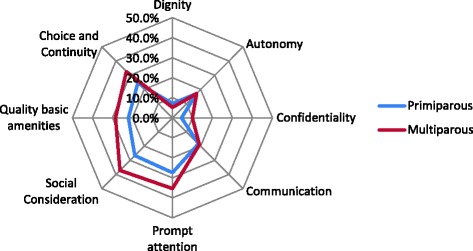
A comparison of the pattern of responsiveness quality for antenatal and birth phases: (**a**) by parity, (**b**) by etnicity, (**c**) by privilege of neighboorhood, (**d**) by perinatal health care pathway, and (**e**) by admission of the child

Figure 1 compares responsiveness performance between advantaged and disadvantaged subpopulations. In all disadvantaged subpopulations the proportion of poor responsiveness was lower. Multiparous women tended to show poorer responsiveness outcomes on nearly all domains. The same pattern was found in women with an obstetric history (see Additional file 1). Ethnic differences were mainly observed within the antenatal phase where Dutch women showed poorer responsiveness outcomes. Women living in a deprived neighbourhood and those who did not speak Dutch proficiently tended to have the same responsiveness pattern (see Additional file 1). Groupings by neighbourhood showed no marked differences for the antenatal phase, while the delivery phase had marked difference in responsiveness outcomes. The nature of the differences in patterns between subgroups are mainly observed for the ‘client orientation’ domains.

**Fig. 2:**
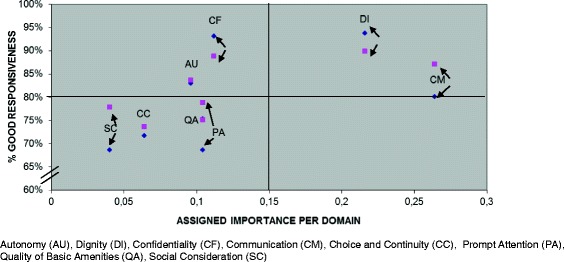
Comparison of the importance assigned to the responsiveness domains and the performance of domains: antenatal and birth phases

The relationship between the proportion of good domain performance and importance was roughly linear as seen in Fig. 2. Of the domains of high importance, average good performance in the Communication domain was above 80% (only 20% rating poor) but better in delivery versus antenatal phases. Of the domains of medium importance, Prompt Attention had low performance (more than 20% rating poor responsiveness), and performance differed widely between antenatal (poorer responsiveness) and birth phases.

Table 4 shows the odds ratios from the multiple logistic regression analyses for responsiveness outcomes and background characteristics, by domain, stratified by phase..Overall, health service characteristics were stronger predictors of responsiveness outcome performance than users’ personal characteristics. In particular, health service characteristics were strong predictors in ‘client orientation’ domain regressions for delivery and birth phases. No background characteristic was significantly associated with ‘respect of person’ domains in the birth phase.

**Table 4:** Variance of reported poor outcome given for each domain for both the antenatal and birth phase. Only Odds Ratio’s (95% CI) for significant determinants are given

Domain	Antenatal phase					Delivery phase				
	Determinants	OR	95% CI		*p*- value	Determinants	OR	95% CI		*p*-value
RESPECT FOR PERSONS						RESPECT FOR PERSONS				
Autonomy	Intervention	3.00	1.44	6.26	0.003	None				
Dignity	None					None				
Communication	None					None				
Confidentiality	Parity	0.33	0.12	0.87	0.025	None				
CLIENT ORIENTATION						CLIENT ORIENTATION				
Choice and Continuity	Ethnic background	0.39	0.20	0.79	0.008	Parity	0.25	0.10	0.62	0.003
Prompt Attention	Obstetric history	2.34	1.08	5.04	0.030	Obstetric history	4.11	1.54	10.99	0.005
	Intervention	2.42	1.14	5.11	0.021	Hospital Admission Child	3.21	1.09	9.49	0.035
						Intervention	2.98	1.17	7.59	0.022
Quality of Basic Amenities	Maternal age	2.10	1.05	4.19	0.036	None				
Social Consideration	Parity	0.42	0.20	0.86	0.018	Obstetric history	2.44	1.05	5.67	0.038
	Ethnic background	0.27	0.13	0.57	0.001	Hospital Admission Child	3.23	1.22	8.54	0.018

Inclusion *p* < 0.05; exclusion *p* < 0.05

Specific domain-background characteristics associations for the antenatal and birth phases were as follows. Higher odds of Prompt Attention problems in both antenatal and birth phases was associated with obstetric history, and having an intervention. Hospital admission of the child was significant in the birth phase only. For Choice and Continuity and Social Consideration in the antenatal phase, having a non-Dutch background (ethnicity) was associated with lower odds of responsiveness problems (OR range: 0.27–0.42). In the birth phase, for Choice and Continuity, only respondents with parity had significantly reduced odds of problems (OR 0.25). Whereas, for Social Consideration obstetric history and hospital admission were associated with higher odds of responsiveness problems (OR range:2.44, 3.23). For Quality of Basic Amenities, only increased maternal age was significantly associated with higher odds of poor responsiveness in the antenatal phase.

## Discussion

Responsiveness quality of perinatal health services in the Netherlands, was better for ‘respect of persons’ domains compared with ‘client orientation’ domains. These are also domains that have more importance to users. Overall, the health status and health-care related characteristics of users explained more of the variation in responsiveness quality than personal characteristics (e.g. education, deprived neighbourhood) in the birth phase, while in the antenatal phase responsiveness is more associated with personal background characteristics.

We observed poorer responsiveness outcomes for the ‘client orientation’ domains, than for the ‘respect of persons’ domains. Similar results were found by Liabsuetrakul et al. and Qing Luo et al. [31, 32] This might be, because the domains of Autonomy, Dignity, Communication and Confidentiality are easier to change. They could be influenced by professionals changing behaviour over short periods of time instead of changes in the organization of care required for ‘client orientation’ domains, which requires management coordination and longer time periods to implement changes. A second explanation might be that the domains in this category are judged as more important by the health professionals and thus are given more attention.

On the whole, there was more variation in responsiveness explained by health-care and health related issues. Obstetric history and an adverse events (receiving an intervention) influenced responsiveness outcomes in the antenatal and birth phases. Other associated personal characteristics were also more health related – maternal age, parity. This is partially in line with reports from the Consumer Assessment of Healthcare Providers and Systems (CAHPS) patient experience survey, which also showed the effect of health-related characteristics. These reports observed more association between age, general health, education, individual health plan, and less association of ethnicity, gender and time in insurance plan with responses on patient experience [33]. Although we did not assess the impact of health plan and length of time in insurance plan, we observed similar these associations for the importance of health and age (since age and parity are inversely related).

Other studies that assessed patient personal characteristics on (some) of the WHO responsiveness domains showed similar tendencies for the characteristics of parity, education and marital status. However these studies did not include birth outcomes within their analysis [31, 34–36]. One could only speculate to what extent differences are explained by cultural factors. More research on this area is needed.

Referral is a common feature of health care systems, in particular with the field of perinatal care. Being referred during pregnancy does not seem to impact responsiveness. This is in line with other studies, which found no association with being referred and responsiveness domains [19, 21]. However, some studies do find a negative association with being referred and patient satisfaction [29].

The domains of Communication and Dignity were most frequently identified as most important. This is partly in contrast with the population based survey conducted by WHO [37] and results by Liabsuetrakul et al. who assessed the importance of responsiveness domains in Thailand [31]. They both found Prompt Attention and Dignity to be the most important domains, followed by Communication in third place. The preference for Prompt Attention in these other studies may be due to the fact that it was operationalized in terms of geographical access and access in case of emergencies. In our study Prompt Attention focussed upon waiting times. Results from other studies which also focussed upon waiting times support our results that Prompt Attention was valued as less important [2, 3]. Qing Luo et al. observed the domains Basic Amenities, Communication and Autonomy to be most important in community health services in China. They reasoned that Prompt Attention was well achieved and therefore not chosen as most important [32]. Bramesfeld et al. saw a similar rankings as ours, but observed a difference in ranking between in- and outpatient mental care. Hereby, observing Prompt Attention to be more important in outpatient care [38].

The overall linear relationship we observed between good domain performance and assigned importance by users may be explained by health care professionals also judging these domains as important, and therefore placing more emphasis on them.

Our study had several strengths. Firstly, 66% of the invited women agreed to participate in this study. This is an effective study sample, since a response rate of 30% has been proposed as reasonable for patient satisfaction surveys and a response rate of 50% is considered to be quite high [13, 14].

Secondly, our study covered many subpopulations in Rotterdam, including subpopulations which are often missed in satisfaction surveys. More frequent among non-participants in satisfaction studies are those having a language barrier, a psychiatric history, a low social economic status, a low educational level, no paid work and Muslim people [39, 40]. Since our study covered these subpopulations, its generalizability to women in perinatal care is more assured. Thirdly, interviews were conducted in such a way that known factors influencing respondent’s health responsiveness outcomes were diminished as much as possible. Interviews were performed by independent interviewers, respondents were interviewed at their own homes and at an appropriate interval with respect to their birth experience(2 weeks postpartum). Previous studies have shown that women who answer surveys at home are more critical compared with respondents who are interviewed in the hospital, since the latter are loyal to the institution [41]. Women being interviewed after 2 weeks also tend to be more critical [42].

A few limitations merit discussion. Firstly, since only people from urban areas participated in this study, the study population is presumably representative for Dutch urban areas, but the generalizability to the whole Dutch perinatal population remains uncertain. Secondly, translation could only be arranged for some of the women who did not understand the Dutch language sufficiently, this was done by a family member of the women. This could introduce a translation bias since this was not done by a professional translator. Thirdly, all non-Dutch ethnic groups were grouped resulting in a heterogeneous subpopulation. Responsiveness outcomes in these subpopulation may differ, since other studies showed that ethnicity can be of influence [22]. Fourthly, no analysis was performed on non-participants. Fifthly, recall bias and carry over effects on health responsiveness outcomes within the antenatal phase cannot be excluded, since birth outcome determinants significantly influenced outcomes within the antenatal phase. Sixtly, we collected medical outcomes from the respondents themselves (self-report), which could lead to less accurate outcomes. In the future one could consider linking the survey data to the medical records. Lastly, the study was slightly underpowered given the power calculation.

## Conclusions

Overall, our ReproQ questionnaire, which was directly derived from the WHO concept of responsiveness, was able to measure responsiveness outcomes of the perinatal care system in the Netherlands. As carry over effects on health responsiveness outcomes within the antenatal phase cannot be excluded we recommend that when evaluating the responsiveness outcomes of the perinatal health care system, antenatal care should be evaluated before the start of delivery to prevent carry over effects of birth outcomes. To improve responsiveness quality of the Dutch Perinatal Care system, caregivers should focus on domains covering the category ‘client orientation’.

## Additional file

Additional file 1: Appendix table. (XLSX 41 kb)

## Abbreviations

CAHPS: Consumer Assessment of Healthcare Providers and Systems
ReproQ: Responsiveness in Perinatal and Obstetric Health Care Questionnaire
WHO: World Health Organization
